# Functional Correlates of Striatal Dopamine Transporter Cerebrospinal Fluid Levels in Alzheimer’s Disease: A Preliminary ^18^F-FDG PET/CT Study

**DOI:** 10.3390/ijms24010751

**Published:** 2023-01-01

**Authors:** Riccardo Camedda, Chiara Giuseppina Bonomi, Martina Gaia Di Donna, Agostino Chiaravalloti

**Affiliations:** 1 Department of Biomedicine and Prevention, University of Rome Tor Vergata, 00133 Rome, Italy; 2 Memory Clinic, Policlinico Tor Vergata, University of Rome, 00133 Rome, Italy; 3 IRCCS Neuromed, 86077 Pozzilli, Italy

**Keywords:** FDG-PET, DAT, Alzheimer’s disease, dopaminergic impairment, dementia

## Abstract

The aim of our study was to investigate regional glucose metabolism with 18F-FDG positron emission tomography/computed tomography in a population of patients with Alzheimer’s disease (AD) in relation to cerebrospinal (CSF) levels of striatal dopamine transporter (DAT). All patients underwent lumbar puncture and received a biomarker-based diagnosis of AD. Differences in regional brain glucose metabolism were assessed by Statistical Parametric Mapping version 12 with the use of age, gender, and MMSE as covariates in the analysis. A positive correlation between CSF DAT levels and glucose metabolism at the level of two brain areas involved in the pathophysiological process of Alzheimer’s disease, the substantia nigra and the posterior cingulate gyrus, has been highlighted. Results indicate that patients with higher CSF DAT levels have a better metabolic pattern in two key zones, suggesting less advanced disease status in patients with more conserved dopaminergic systems.

## 1. Introduction

Alzheimer’s disease (AD) is a progressive and incurable neurological disorder with complex etiology leading to cognitive decline and dementia. The main histopathological hallmarks are extracellular accumulation of neuritic plaques of amyloid-ꞵ (Aꞵ) peptides and intracellular deposition of tau protein aggregates, which cause progressive synaptic and neuronal damage that primarily affects the hippocampus and cerebral cortex [1,2,3,4,5,6,7].

Scientific advances allow us to study these alterations in vivo, and brain glucose hypometabolism, measured with [^18^F]-FDG PET, is held as a marker of neurodegeneration in AD. This technique measures regional glucose consumption related to local glutamatergic synaptic and astrocyte activity, identifying the location and extension of areas of hypometabolism reflecting local neuronal dysfunction [8,9].

Numerous studies have taken place over the years, allowing us to identify, with good certainty, brain areas that show early reduction in glucose metabolism even before clinically evident AD and, likewise, those areas which are later involved or even spared during disease course [10,11,12,13].

The involvement of subcortical structures is also an important player in AD, which may be reflected by findings of altered levels of dopamine (DA) [14], possibly responsible for the psycho-behavioral alterations typical of the disease, such as depression and apathy. Over a third of AD patients can also develop extrapyramidal signs, supporting the presence of degenerative changes in dopamine-containing neurons [15]. However, motor symptoms are usually present only in late stages of the disease, while recent literature highlights an early involvement of dopaminergic transmission in AD, determining cognitive impairment itself [16,17,18,19,20,21,22].

At the synaptic level, DA levels are regulated by the dopamine transporter (DAT), a transmembrane sodium chloride-dependent protein selectively expressed on the presynaptic membrane of dopaminergic cells. It is responsible for dopamine reuptake, thus terminating its activity in the synapse [23,24,25], and modulates quantal DA release at endplates, regulating also its storage within synaptic vesicles [26].

The expression of DAT on presynaptic terminals reflects striatal dopaminergic innervation, implying a direct relationship between its reduction and dopaminergic cell loss [27]. Cerebrospinal (CSF) levels of DAT can reflect its synaptic levels and, therefore, can be held as a proxy of the dopaminergic function throughout the whole cortex.

Our study fits into this context, with the role of the dopaminergic system still at the center of the scientific debate to clarify its involvement in the pathogenesis of Alzheimer’s disease, especially in the earliest stages of the disease.

We aimed to investigate the possible bond between CSF levels of DAT and regional brain metabolism studied with 18F-FDG positron emission tomography/computed tomography (PET/CT) in a population of patients with AD, in order to deepen the knowledge on the role of dopaminergic degeneration in the pathogenesis of the disease.

## 2. Results

Our study documented a correlation between CSF levels of DAT and regional brain metabolism measured with 18F-FDG PET/CT in patients with AD.

**Figure 1:**
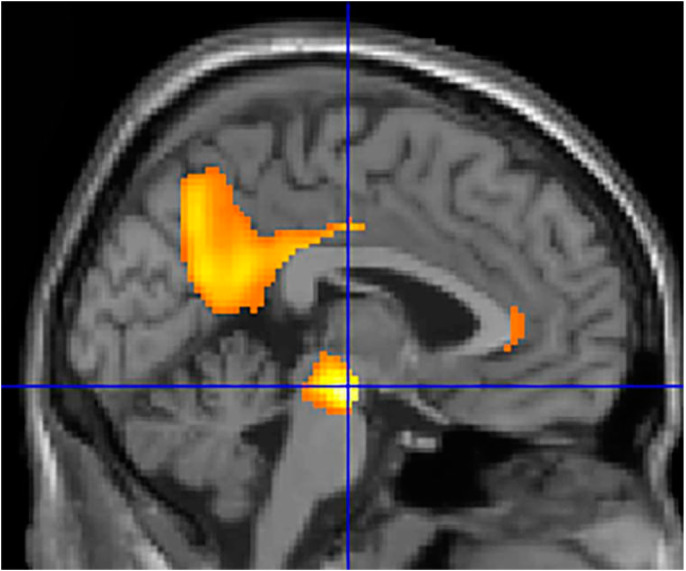
Magnetic resonance superimposition of the data presented in Table 1 showing an increased glucose consumption in the substantia nigra and in the posterior cingulate cortex (sagittal view).

In particular, the analysis of the data shows that within the AD population a higher CSF value of DAT is associated with a relative less glucidic hypometabolism at the level of the substantia nigra and the Brodmann area 31, known as dorsal posterior cingulate area. Detailed results are provided in Table 1 and Figure 1.

## 3. Discussion

Although very debated, the involvement of the dopaminergic system in the pathophysiology of AD is at the center of the scientific debate, with the latest studies seemingly confirming this hypothesis. Notably, although it has been recently demonstrated that the dopaminergic system is altered predominantly within the mesocorticolimbic system [19], several findings also partially support the involvement of the nigrostriatal pathway [28]. In line with this, in the present work we found a significant association between CSF DAT levels and both glucose metabolism of the SN, starting hub of the nigro-striatal pathway, and of the PCC, key hub of the DMN with an early and specific vulnerability in AD pathology. It is possible that CSF DAT values are representative of the good functionality of both pathways.

Indeed, dopaminergic dysfunction may be linked to lower DAT levels, as suggested by a study showing reduced dopamine release in mice with low DAT [29]. Over time, several studies have been conducted with different results regarding the alterations of DAT in AD. DAT levels decline by age 50, suggesting an age-related reduction of its levels [30]. Although some studies show no differences in DAT levels between AD and control, several papers reported reduced levels of DAT synthesis in AD compared to controls [31,32].

Some neuroimaging studies have partially supported these data, showing dopaminergic alterations in the striatum and hippocampus in patients with overt AD dementia. In one paper, [^11^C]β-CFT, a cocaine analogue, was used as a radioligand to investigate striatal dopamine reuptake sites in AD patients by PET method, and a reduction of about 20% of radioligand uptake in both the putamen and the caudate nuclei was identified in these patients compared to controls. Furthermore, the greater the reduction in tracer fixation in these areas, the more severe the extrapyramidal symptoms [33].

The striatum, with its portions represented by the caudate and putamen nuclei, is the arrival station of the fibers coming from the pars compacta of the substantia nigra (SN), the nigro-striatal pathway [34,35]. The SN is a midbrain dopaminergic nucleus that has a critical role in modulating motor movement and reward functions as part of the basal ganglia circuitry.

These are a multiplicity of interconnected subcortical nuclei that govern numerous bodily functions, from voluntary movement, cognitive planning, emotion and reward function, and even cognition. The SN is considered the primary input of the basal ganglia circuits and consequently an element of fundamental importance for all regulated functions [36], and can be functionally and morphologically divided into two portions, the pars compacta characterized by the prevalent presence of dopaminergic neurons and the pars reticulata with the prevalent presence of inhibitory GABAergic neurons [37].

The nigro-striatal pathway, in synergy with the cortico-striatal glutamatergic projections, is responsible for the control of voluntary movements [34,35], critically involved in the extrapyramidal motor deficits typical of Parkinson’s disease [36].

The prevalence of motor symptoms in AD increases with disease progression [38]. Although the underlying neuropathological substrate has not yet been fully elucidated, several clinicopathological studies indicate that the onset of extrapyramidal signs in AD may be related to heterogeneous pathologies involving the substantia nigra and the related extrapyramidal system [38,39,40].

Alterations of the SN are frequently present in AD and the involvement of the dopaminergic system has been confirmed by some post-mortem studies which have shown alterations at the level of the midbrain, specifically in the SN itself and in the ventral tegmental area (VTA) [41,42,43,44,45]. The latter represents the beginning of the mesocortical–limbic pathway, directed to the hippocampus, cerebral cortex, and the nucleus accumbens; dopaminergic terminals along with glutamatergic projections from the amygdala, hippocampus, and prefrontal cortex, are involved in the control of volition and reward [28,46].

Neurons of these areas, involved in the formation of the nigrostriatal and meso-cortico-limbic pathways, show typical histopathological changes such as the presence of NFT, surrounding Aβ deposition, neuropil threads, cellular death, and also a decrease in DA content, suggesting an active role of dopaminergic alterations in the pathophysiology of AD [15,42,45,47,48,49,50,51].

Neuronal loss is known to be associated with zonal hypometabolism in [^18^F]FDG-PET images. This method allows an in vivo evaluation of the regional brain glucose metabolism, which is a proxy of synaptic function and density [52,53,54].

Numerous studies conducted over the years have confirmed this, agreeing in noting a constant reduction in brain glucose metabolism in the areas most characterized by atrophy and neuronal loss in subjects with Alzheimer’s disease. In particular, the initial stages of the disease are distinguished by a glucose hypometabolism predominantly at the level of the precuneus, the posterior cingulate, and the temporal and parietal cortical areas while, as the disease progresses, hypometabolism extends to the frontal cortex to affect the entire brain, sparing only some specific areas [10,11,12,13].

The relative local brain hypermetabolism investigated with the [^18^F]FDG-PET method at the level of the SN identified in our study in AD patients with higher CSF DAT levels suggests a relative well-being of the specific area, less affected by degenerative processes and neuronal loss. The minor involvement of the substantia nigra in neurodegenerative processes is a result in agreement with the higher CSF DAT levels, indicating relative conservation of dopaminergic function at the level of striatal circuits.

Conversely, assuming a progressive decrease of DAT in AD, we could speculate the presence of a parallel alteration of the dopaminergic functions at the level of striatal circuits [26,29].

The well-being of the nigro-striatal pathway might be associated with a lower incidence of extrapyramidal motor deficits, typical of Parkinson’s disease, but also present in AD in its more advanced stages [36,38]. None of the AD patients in our study had extrapyramidal signs, but a vulnerability of the extrapyramidal system cannot be excluded and longitudinal follow-ups are needed to evaluate the future appearance of movement disorders.

The suggestion of an earlier disease stage in patients with higher CSF DAT levels may also be supported by the second result of our study, the finding in these patients of a relative less glucidic hypometabolism at the level of the dorsal portion of the posterior cingulate gyrus, corresponding to the Brodmann area 31.

The posterior cingulate cortex (PCC) is a highly anatomically and functionally connected region in the brain that represents an important hub in the default mode network [55,56], which is early disrupted in AD [57,58,59]. Decreased uptake of 18F-FDG in the posterior cingulate cortex (PCC) is a specific feature of AD [60,61], and is established as an important discriminating signature feature to distinguish AD from other dementias.

Indeed, PCC hypometabolism has been shown to be highly sensitive and specific even in the early stages of the disease, and thus forms the core of the FDG-PET hypometabolic pattern of AD [62,63,64,65].

It is known that a progressive reduction of regional cerebral glucose metabolism in specific areas occurs years before the onset of clinical symptoms in patients with AD [65,66,67,68,69]. Of all the areas typically involved in pathology, the posterior cingulate cortex appears to represent the most sensitive marker for predicting which patients will progress from a state of MCI to AD [65,69,70].

The finding of relatively more conserved glucose metabolism at the level of the posterior cingulate gyrus in patients with higher CSF DAT levels leads us to hypothesize a less advanced disease status in patients with a more conserved dopaminergic system as if to suggest a protective role of the latter against the progression of the disease.

In contrast to what has just been hypothesized, a second interpretation could correlate the increased uptake of FDG found at substantia nigra and posterior cingulate gyrus level in patients with higher CSF DAT levels to an inflammatory state of these areas.

Although the involvement of neuroinflammatory processes in Alzheimer’s disease is known, the actual role in the pathogenesis has not yet been clarified and is being studied [71,72,73,74]; advances in molecular imaging using PET have provided insights into the time course of neuroinflammation and its relation to Alzheimer’s disease.

A recent study has shown that in AD brains the absorption of FDG and TSPO (the most widely investigated neuroinflammation target for PET imaging [75]) are correlated, with higher levels in the more conserved regions. Furthermore, the FDG signal is reduced in proportion to the neuronal loss in the most affected regions, losing correlation with TSPO.

These results suggest that both neurons and microglia contribute to the FDG signal in neurodegenerative diseases with their relative contribution depending on how advanced the disease is in each brain region.

In the early stages, with a predominantly healthy brain, the FDG signal of microglia predominates, which can mask the initial decline in neuronal metabolism; the latter becomes predominant in the advanced stages [76].

In consideration of this, the patients in which a greater uptake of FDG was found in the substantia nigra and in the posterior cingulate cortex would represent the part of the population in an earlier pathophysiological stage, with the high carbohydrate content sustained by microglial activation to compensate for the initial neuronal loss and the high CSF DAT levels confirming a still poorly compromised dopaminergic system.

This hypothesis should be confirmed with new PET studies that use specific tracers for neuroinflammation and correlate CSF levels of DAT to the PET fixation of these tracers in specific brain areas.

We are aware that this study has several limitations. Specifically, it could be considered a preliminary study, conducted within a single AD population; to acquire greater value, it should be replicated in the future by comparing the values of local cerebral glucose metabolism of AD patients with those of a population of healthy individuals.

Furthermore, the sample under study should be numerically enlarged in order to obtain more significant data, and follow-up longitudinal clinical data could be implemented to explore the relationship between our findings and the development of extrapyramidal signs in AD.

## 4. Materials and Methods

### 4.1. Subjects’ Enrolment

The study retrospectively included 28 consecutive patients evaluated at the UOSD Centro Demenze of the University Hospital “Policlinico Tor Vergata” in Rome between December 2020 and June 2022. After a complete diagnostic work-up—including medical history, neurological examination, blood screening, neuropsychological assessment, magnetic resonance imaging and lumbar puncture—all patients received a biomarker-based diagnosis of AD according to the NIA-AA research framework [77]. All selected patients had previously undergone PET/CT brain scan as part of the diagnostic assessment at the Nuclear Medicine Unit of the same facility.

Exclusion criteria were history of manifest acute stroke—Hachinski scale score > 4 or radiological evidence of focal ischemic lesions-, the presence of another diagnosed neurodegenerative or psychiatric disorder, signs of extrapyramidal disorder, and treatment with antipsychotic or antiparkinsonian drugs. No patient was treated with memantine or acetylcholinesterase inhibitors until the end of the screening. Demographics from the study cohort are reported in Table 2.

All participants or their legal guardian provided written informed consent after receiving an extensive description of the study. The study was conducted according to the Declaration of Helsinki.

### 4.2. CSF Collection and Analysis

All lumbar punctures were performed with sterile technique between 8 and 10 a.m., a total of 10 mL per patient CSF sample was collected in polypropylene tubes. A measurement of 2 mL of all samples was used for biochemical routine analysis, including cell and protein count, the remaining sample was centrifuged at 2000 g at +4 °C for 10 min, aliquoted in 1 mL portions, and frozen at −80 °C for further analysis of levels of Aβ_42_, p-tau phosphorylated at Thr181, t-tau, and DAT dosing. Blood samples were also drawn for complementary analysis.

Commercially available kits were used to carry out biochemical analysis (Flex reagent cartridge, Dimension Vista System, Siemens Healthcare Diagnostics GmbH, Munich, Germany); CSF Aβ_42_, p-tau, and t-tau were determined using a sandwich enzyme-linked immunosorbent assay (EUROIMMUN Aβ1-42 levels ELISA^©^, EUROIMMUN Total tau ELISA^©^, EUROIMMUN p-Tau (181) ELISA^©^). DAT CSF contents were determined using Luminex Multiplex Assay (Thermo Fisher Scientific, Waltham, MA, USA), according to the manufacturer’s instructions. Concentration of analytes was calculated according to a standard curve and expressed as picograms per milliliter.

### 4.3. ^18^F-FDG PET Scanning Protocol

[^18^F]FDG PET/CT brain scan was performed after overnight fasting (at least 6 h). I.v. radiopharmaceutical injection ([^18^F] FDG) was performed after checking the blood glucose values, to ensure they were within the range proposed by the guidelines of the European Association of Nuclear Medicine. All subjects were injected intravenously with 185–215 MBq of [^18^F]FDG and were hydrated with 500 mL of NaCl 0.9%. The PET system Discovery VCT (GE Medical System—PET/CT scanner) was used to assess [^18^F]FDG brain distribution in all patients. PET/CT scanner acquisition was started 30 ± 5 min after [^18^F] FDG injection and lasted 10 min in all the subjects. The following reconstruction parameters have been set: ordered subsets expectation maximization, 4 subsets and 12 iterations; matrix 256 × 256; full width at half maximum (FWHM): 5 mm.

### 4.4. Statistical Analysis

Statistical parametric mapping 12 (SPM12) implemented in Matlab 2018a was used for the analysis of PET scans in this study (https://www.fil.ion.ucl.ac.uk/spm/software/spm12/ - accessed on 27 December 2022). PET data were converted from DICOM to Nifti format using Mricron software v 1.0.20190902 available at https://www.nitrc.org/projects/mricron – accessed on 27 December 2022 - and then subjected to a normalization process. A bias regularization was applied (0.0001) in order to limit biases due to smooth, spatially varying artifacts that modulate the intensity of the image and that can impede the automating processing of the images. FWHM of Gaussian smoothness of bias (to prevent the algorithm from trying to model out intensity variation due to different tissue types) was set at 60 mm cutoff; tissue probability map implemented in SPM12 was used (TPM.nii). A mutual information affine registration with the tissue probability maps was used to achieve approximate alignment to ICBM space template—European brains [78,79]. Warping regularization was set with the following 1 by 5 array (0, 0.001, 0.5, 0.05, 0.2); smoothness (to cope with functional anatomical variability that is not compensated by spatial normalization and to improve the signal to noise ratio) was set at 5 mm; sampling distance (that encodes the approximate distance between sampled points when estimating the model parameters) was set at 3.

We applied an 8 mm isotropic Gaussian filter to blur the individual variations (especially gyral variations) and to increase the signal-to-noise ratio. We used the following parameters and post-processing tools before regression analysis was applied: global normalization (that escalates images to a global value) = 50 (using proportional scaling); masking threshold (that helps to identify voxels with an acceptable signal in them) was set to 0.8; transformation tool of statistical parametric maps into normal distribution; correction of SPM coordinates to match the Talairach coordinates, subroutine implemented by Matthew Brett (http://www.mrc-cbu.cam.ac.uk/Imaging, accessed on 27 December 2022). Brodmann areas (BA) were identified at a range from 0 to 3 mm from the corrected Talairach coordinates of the SPM output isocenter by using a Talairach client available at http://www.talairach.org/index.html, accessed on 27 December 2022. As proposed by Bennett et al. [80], SPM t-maps have been corrected for multiple comparisons with the false discovery rate (*p* ≤ 0.05) and corrected for multiple comparisons at the cluster level (*p* ≤ 0.001). The level of significance was set at 100 (5 × 5 × 5 voxels, i.e., 11 × 11 × 11 mm) contiguous voxels. We performed a regression analysis with CSF DAT values, using a ‘multiple regression’ design model available in SPM12 as in another similar report of our group in this field [81]. We used sex, age, and MMSE as covariates in the analyses.

## 5. Conclusions

The results of our study suggest that the positive correlation found between CSF DAT levels and cerebral glucose metabolism studied with FDG-PET at the level of the substantia nigra and posterior cingulate gyrus may indicate a less advanced stage of disease in subjects with a dopaminergic system less compromised. The hypothesis can be advanced both by considering the better glucose content of the two brain areas mentioned as an expression of a degenerative process still in the initial stages, and by associating the relative hypermetabolism with phenomena of an inflammatory nature, with the FDG signal of activated microglia which predominates in the initial stages of the disease, masking the initial decline in neuronal metabolism.

## Figures and Tables

**Table 1:** Regression analysis showing the CSF DAT levels-related areas of increased 18F-FDG brain uptake.

Analysis		Cluster Level				Voxel Level		
Cluster P		Cluster	Cluster	Cortical	Z Score of	Talairach	Cortical Region	BA
(FWE-Corr)		p(FDR-Corr)	Extent	Region	Maximum	Coordinates		
Positive correlation	0.000	0.000	21,570	Mid Brain	3.65	−6, −16, −14	Substantia Nigra	
				R Limbic	3.43	16, −36, 40	Posterior cingulate	31

In the ‘cluster level’ section on left, the number of voxels, the corrected *p* value of significance and the cortical region where the voxel is found, are all reported for each significant cluster. In the ‘voxel level’ section, all of the coordinates of the correlation sites (with the Z-score of the maximum correlation point), the corresponding cortical region and BA are reported for each significant cluster. BA: Brodmann’s area. In the case that the maximum correlation is achieved outside the gray matter, the nearest gray matter (within a range of 5 mm) is indicated with the corresponding BA. L, left; R, right; FWE: familywise error; FDR: false discovery rate.

**Table 2:** Demographic data from the study cohort.

	Study Cohort (*n* = 28)
Age (yo)	70.64 ± 6.16
Sex (M:F)	13:15
CSF Aβ42 (pg/mL)	379.07 ± 98.02
CSF p-tau (pg/mL)	68.29 ± 27–28
CSF t-tau (pg/mL)	501.61 ± 251.82
CSF DAT (pg/mL)	2.86 ± 1.32

DAT: Dopamine transporter; p-Tau: phosphorylated tau; t-Tau: total tau.

## References

[1] Villemagne V.L., Burnham S., Bourgeat P., Brown B., Ellis K.A., Salvado O., Szoeke C., Macaulay S.L., Martins R., Maruff P. (2013). Australian Imaging Biomarkers and Lifestyle (AIBL) Research Group. Amyloid β deposition, neurodegeneration, and cognitive decline in sporadic Alzheimer’s disease: A prospective cohort study. Lancet Neurol..

[2] DeTure M.A., Dickson D.W. (2019). The neuropathological diagnosis of Alzheimer’s disease. Mol. Neurodegener..

[3] Alzheimer A., Stelzmann R.A., Schnitzlein H.N., Murtagh F.R. (1995). An English translation of Alzheimer’s 1907 paper, “Uber eine eigenartige Erkankung der Hirnrinde”. Clin. Anat..

[4] Hardy J., Revesz T. (2012). The spread of neurodegenerative disease. N. Engl. J. Med..

[5] D′Amelio M., Rossini P.M. (2012). Brain excitability and connectivity of neuronal assemblies in Alzheimer’s disease: From animal models to human findings. Prog. Neurobiol..

[6] Roy D.S., Arons A., Mitchell T.I., Pignatelli M., Ryan T.J., Tonegawa S. (2016). Memory retrieval by activating engram cells in mouse models of early Alzheimer’s disease. Nature.

[7] Scheff S.W., Price D.A., Schmitt F.A., Mufson E.J. (2006). Hippocampal synaptic loss in early Alzheimer’s disease and mild cognitive impairment. Neurobiol. Aging.

[8] Zimmer E.R., Parent M.J., Souza D.G., Leuzy A., Lecrux C., Kim H.I., Gauthier S., Pellerin L., Hamel E., Rosa-Neto P. (2017). [18F]FDG PET signal is driven by astroglial glutamate transport. Nat. Neurosci..

[9] Frisoni G.B., Boccardi M., Barkhof F., Blennow K., Cappa S., Chiotis K., Démonet J.F., Garibotto V., Giannakopoulos P., Gietl A. (2017). Strategic roadmap for an early diagnosis of Alzheimer’s disease based on biomarkers. Lancet Neurol..

[10] Mosconi L., Mistur R., Switalski R., Tsui W.H., Glodzik L., Li Y., Pirraglia E., De Santi S., Reisberg B., Wisniewski T. (2009). FDG-PET changes in brain glucose metabolism from normal cognition to pathologically verified Alzheimer’s disease. Eur. J. Nucl. Med. Mol. Imaging.

[11] Alexander G.E., Chen K., Pietrini P., Rapoport S.I., Reiman E.M. (2002). Longitudinal PET Evaluation of Cerebral Metabolic Decline in Dementia: A Potential Outcome Measure in Alzheimer’s Disease Treatment Studies. Am. J. Psychiatry.

[12] Minoshima S., Frey K.A., Koeppe R.A., Foster N.L., Kuhl D.E. (1995). A diagnostic approach in Alzheimer’s disease using three-dimensional stereotactic surface projections of fluorine-18-FDG PET. J. Nucl. Med..

[13] Herholz K., Carter S.F., Jones M. (2007). Positron emission tomography imaging in dementia. Br. J. Radiol..

[14] Henjum K., Watne L.O., Godang K., Halaas N.B., Eldholm R.S., Blennow K., Zetterberg H., Saltvedt I., Bollerslev J., Knapskog A.B. (2022). Cerebrospinal fluid catecholamines in Alzheimer’s disease patients with and without biological disease. Transl. Psychiatry.

[15] Lopez O.L., Wisnieski S.R., Becker J.T., Boiler F., DeKosky S.T. (1997). Extrapyramidal signs in patients with probable Alzheimer disease. Arch. Neurol..

[16] Storga D., Vrecko K., Birkmayer J.G., Reibnegger G. (1996). Monoaminergic neurotransmitters, their precursors and metabolites in brains of Alzheimer patients. Neurosci. Lett..

[17] Trillo L., Das D., Hsieh W., Medina B., Moghadam S., Lin B., Dang V., Sanchez M.M., De Miguel Z., Ashford J.W. (2013). Ascending monoaminergic systems alterations in Alzheimer’s disease. Translating basic science into clinical care. Neurosci. Biobehav. Rev..

[18] Koch G., Di Lorenzo F., Bonnì S., Giacobbe V., Bozzali M., Caltagirone C., Martorana A. (2014). Dopaminergic modulation of cortical plasticity in Alzheimer’s disease patients. Neuropsychopharmacology.

[19] Palmer K., Di Iulio F., Varsi A.E., Gianni W., Sancesario G., Caltagirone C., Spalletta G. (2010). Neuropsychiatric predictors of progression from amnestic-mild cognitive impairment to Alzheimer’s disease: The role of depression and apathy. J. Alzheimers Dis..

[20] Sala A., Caminiti S.P., Presotto L., Pilotto A., Liguori C., Chiaravalloti A., Garibotto V., Frisoni G.B., D’Amelio M., Paghera B. (2021). In vivo human molecular neuroimaging of dopaminergic vulnerability along the Alzheimer’s disease phases. Alzheimers Res. Ther..

[21] Martorana A., Koch G. (2014). Is dopamine involved in Alzheimer’s disease?. Front. Aging Neurosci..

[22] Itoh A., Nitta A., Nadai M., Nishimura K., Hirose M., Hasegawa T., Nabeshima T. (1996). Dysfunction of cholinergic and dopaminergic neuronal systems in beta-amyloid protein—infused rats. J. Neurochem..

[23] Cheramy A., Leviel V., Glowinski J. (1981). Dendritic release of dopamine in the substantia nigra. Nature.

[24] Nissbrandt H., Sundström E., Jonsson G., Hjorth S., Carlsson A. (1989). Synthesis and release of dopamine in rat brain: Comparison between substantia nigra pars compacts, pars reticulata, and striatum. J. Neurochem..

[25] Uhl G.R. (2003). Dopamine transporter: Basic science and human variation of a key molecule for dopaminergic function, locomotion, and parkinsonism. Mov. Disord..

[26] Sulzer D., Cragg S.J., Rice M.E. (2016). Striatal dopamine neurotransmission: Regulation of release and uptake. Basal Ganglia.

[27] Palermo G., Giannoni S., Bellini G., Siciliano G., Ceravolo R. (2021). Dopamine Transporter Imaging, Current Status of a Potential Biomarker: A Comprehensive Review. Int. J. Mol. Sci..

[28] Haber S.N., Knutson B. (2010). The reward circuit: Linking primate anatomy and human imaging. Neuropsychopharmacology.

[29] Giros B., Jaber M., Jones S.R., Wightman R.M., Caron M.G. (1996). Hyperlocomotion and indifference to cocaine and amphetamine in mice lacking the dopamine transporter. Nature.

[30] Ma S.Y., Ciliax B.J., Stebbins G., Jaffar S., Joyce J.N., Cochran E.J., Kordower J.H., Mash D.C., Levey A.I., Mufson E.J. (1999). Dopamine transporter-immunoreactive neurons decrease with age in the human substantia nigra. J. Comp. Neurol..

[31] Murray A.M., Weihmueller F.B., Marshall J.F., Hurtig H.I., Gottleib G.L., Joyce J.N. (1995). Damage to dopamine systems differs between Parkinson’s disease and Alzheimer’s disease with parkinsonism. Ann. Neurol..

[32] Joyce J.N., Meador-Woodruff J.H. (1997). Linking the family of D2 receptors to neuronal circuits in human brain: Insights into schizophrenia. Neuropsychopharmacology.

[33] Rinne J.O., Sahlberg N., Ruottinen H., Någren K., Lehikoinen P. (1998). Striatal uptake of the dopamine reuptake ligand [11C]beta-CFT is reduced in Alzheimer’s disease assessed by positron emission tomography. Neurology.

[34] Lammel S., Ion D.I., Roeper J., Malenka R.C. (2011). Projection-specific modulation of dopamine neuron synapses by aversive and rewarding stimuli. Neuron.

[35] Bolam J.P., Pissadaki E.K. (2012). Living on the edge with too many mouths to feed: Why dopamine neurons die. Mov. Disord..

[36] Hodge G.K., Butcher L.L. (1980). Pars compacta of the substantia nigra modulates motor activity but is not involved importantly in regulating food and water intake. Naunyn Schmiedebergs Arch. Pharmacol..

[37] Sonne J., Reddy V., Beato M.R. (2022). Neuroanatomy, Substantia Nigra. 2021 Oct 30. StatPearls [Internet].

[38] Morris J.C., Drazner M., Fulling K., Grant E.A., Goldring J. (1989). Clinical and pathological aspects of parkinsonism in Alzheimer’s disease. A role for extranigral factors?. Arch. Neurol..

[39] Kazee A.M., Cox C., Richfield E.K. (1995). Substantia nigra lesions in Alzheimer disease and normal aging. Alzheimer Dis. Assoc. Disord..

[40] Liu Y., Stern Y., Chun M.R., Jacobs D.M., Yau P., Goldman J.E. (1997). Pathological correlates of extrapyramidal signs in Alzheimer’s disease. Ann. Neurol..

[41] Joyce J.N., Smutzer G., Whitty C.J., Myers A., Bannon M.J. (1997). Differential modification of dopamine transporter and tyrosine hydroxylase mRNAs in midbrain of subjects with Parkinson’s, Alzheimer’s with parkinsonism, and Alzheimer’s disease. Mov. Disord..

[42] Gibb W.R., Mountjoy C.Q., Mann D.M., Lees A.J. (1989). The substantia nigra and ventral tegmental area in Alzheimer’s disease and Down’s syndrome. J. Neurol. Neurosurg. Psychiatry.

[43] Mann D.M., Yates P.O., Marcyniuk B. (1984). Monoaminergic neurotransmitter systems in presenile Alzheimer’s disease and in senile dementia of Alzheimer type. Clin. Neuropathol..

[44] Attems J., Quass M., Jellinger K.A. (2007). Tau and alpha-synuclein brainstem pathology in Alzheimer disease: Relation with extrapyramidal signs. Acta Neuropathol..

[45] Burns J.M., Galvin J.E., Roe C.M., Morris J.C., McKeel D.W. (2005). The pathology of the substantia nigra in Alzheimer disease with extrapyramidal signs. Neurology.

[46] Haber S.N., Fudge J.L. (1997). The primate substantia nigra and VTA: Integrative circuitry and function. Crit. Rev. Neurobiol..

[47] Rudelli R.D., Ambler M.W., Wisniewski H.M. (1984). Morphology and distribution of Alzheimer neuritic (senile) and amyloid plaques in striatum and diencephalon. Acta Neuropathol..

[48] Braak H., Braak E. (1990). Alzheimer’s disease: Striatal amyloid deposits and neurofibrillary changes. J. Neuropathol. Exp. Neurol..

[49] Selden N., Mesulam M.M., Geula C. (1994). Human striatum: The distribution of neurofibrillary tangles in Alzheimer’s disease. Brain Res..

[50] Roostaei T., Nazeri A., Felsky D., De Jager P.L., Schneider J.A., Pollock B.G., Bennett D.A., Voineskos A.N. (2017). Alzheimer’s Disease Neuroimaging Initiative (ADNI). Genome-wide interaction study of brain beta-amyloid burden and cognitive impairment in Alzheimer’s disease. Mol. Psychiatry.

[51] Ditter S.M., Mirra S.S. (1987). Neuropathologic and clinical features of Parkinson’s disease in Alzheimer’s disease patients. Neurology.

[52] Sibson N.R., Dhankhar A., Mason G.F., Rothman D.L., Behar K.L., Shulman R.G. (1998). Stoichiometric coupling of brain glucose metabolism and glutamatergic neuronal activity. Proc. Natl. Acad. Sci. USA.

[53] Sokoloff L., Reivich M., Kennedy C., Rosiers M.D., Patlak C.S., Pettigrew K.E.A., Sakurada O., Shinohara M. (1977). The [14C]deoxyglucose method for the measurement of local cerebral glucose utilization: Theory, procedure, and normal values in the conscious and anesthetized albino rat. J. Neurochem..

[54] Laforce R., Soucy J.P., Sellami L., Dallaire-Théroux C., Brunet F., Bergeron D., Miller B.L., Ossenkoppele R. (2018). Molecular imaging in dementia: Past, present, and future. Alzheimers Dement..

[55] Buckner R.L., Andrews-Hanna J.R., Schacter D.L. (2008). The brain’s default network: Anatomy, function, and relevance to disease. Ann. N. Y. Acad. Sci..

[56] Raichle M.E., MacLeod A.M., Snyder A.Z., Powers W.J., Gusnard D.A., Shulman G.L. (2001). A default mode of brain function. Proc. Natl. Acad. Sci. USA.

[57] Jones D.T., Knopman D.S., Gunter J.L., Graff-Radford J., Vemuri P., Boeve B.F., Petersen R.C., Weiner M.W., Jack C.R. (2016). Alzheimer’s Disease Neuroimaging Initiative. Cascading network failure across the Alzheimer’s disease spectrum. Brain.

[58] Raichle M.E. (2015). The brain’s default mode network. Annu. Rev. Neurosci..

[59] Buckner R.L., Snyder A.Z., Shannon B.J., LaRossa G., Sachs R., Fotenos A.F., Sheline Y.I., Klunk W.E., Mathis C.A., Morris J.C. (2005). Molecular, structural, and functional characterization of Alzheimer’s disease: Evidence for a relationship between default activity, amyloid, and memory. J. Neurosci..

[60] Herholz K. (2014). Guidance for reading FDG PET scans in dementia patients. Q. J. Nucl. Med. Mol. Imaging.

[61] Kato T., Inui Y., Nakamura A., Ito K. (2016). Brain fluorodeoxyglucose (FDG) PET in dementia. Aging Res. Rev..

[62] Foster N.L., Heidebrink J.L., Clark C.M., Jagust W.J., Arnold S.E., Barbas N.R., DeCarli C.S., Scott Turner R., Koeppe R.A., Higdon R. (2007). FDG-PET improves accuracy in distinguishing frontotemporal dementia and Alzheimer’s disease. Brain.

[63] Brown R.K., Bohnen N.I., Wong K.K., Minoshima S., Frey K.A. (2014). Brain PET in suspected dementia: Patterns of altered FDG metabolism. Radiographics.

[64] Silverman D.H., Small G.W., Chang C.Y., Lu C.S., de Aburto M.A.K., Chen W., Czernin J., Rapoport S.I., Pietrini P., Alexander G.E. (2001). Positron emission tomography in evaluation of dementia: Regional brain metabolism and long-term outcome. JAMA.

[65] Minoshima S., Giordani B., Berent S., Frey K.A., Foster N.L., Kuhl D.E. (1997). Metabolic reduction in the posterior cingulate cortex in very early Alzheimer’s disease. Ann. Neurol..

[66] Cerami C., Della Rosa P.A., Magnani G., Santangelo R., Marcone A., Cappa S.F., Perani D. (2014). Brain metabolic maps in Mild Cognitive Impairment predict heterogeneity of progression to dementia. Neuroimage Clin..

[67] Jagust W., Gitcho A., Sun F., Kuczynski B., Mungas D., Haan M. (2006). Brain imaging evidence of preclinical Alzheimer’s disease in normal aging. Ann. Neurol..

[68] Salmon E., Lekeu F., Garraux G., Guillaume B., Magis D., Luxen A., Moonen G., Collette F. (2008). Metabolic correlates of clinical heterogeneity in questionable Alzheimer’s disease. Neurobiol. Aging.

[69] Drzezga A., Lautenschlager N., Siebner H., Riemenschneider M., Willoch F., Minoshima S., Schwaiger M., Kurz A. (2003). Cerebral metabolic changes accompanying conversion of mild cognitive impairment into Alzheimer’s disease: A PET follow-up study. Eur. J. Nucl. Med. Mol. Imaging.

[70] Silverman D.H. (2004). Brain 18F-FDG PET in the diagnosis of neurodegenerative dementias: Comparison with perfusion SPECT and with clinical evaluations lacking nuclear imaging. J. Nucl. Med..

[71] Leng F., Edison P. (2021). Neuroinflammation and microglial activation in Alzheimer disease: Where do we go from here?. Nat. Rev. Neurol..

[72] Ransohoff R.M. (2016). How neuroinflammation contributes to neurodegeneration. Science.

[73] Allen N.J., Lyons D.A. (2018). Glia as architects of central nervous system formation and function. Science.

[74] Heneka M.T., Carson M.J., El Khoury J., Landreth G.E., Brosseron F., Feinstein D.L., Jacobs A.H., Wyss-Coray T., Vitorica J., Ransohoff R.M. (2015). Neuroinflammation in Alzheimer’s disease. Lancet Neurol..

[75] Luzi F., Savickas V., Taddei C., Hader S., Singh N., Gee A.D., Bongarzone S. (2020). Radiolabeling of [^11^C]FPS-ZM1, a receptor for advanced glycation end products-targeting positron emission tomography radiotracer, using a [^11^C]CO_2_-to-[^11^C]CO chemical conversion. Future Med. Chem..

[76] Xiang X., Wind K., Wiedemann T., Blume T., Shi Y., Briel N., Beyer L., Biechele G., Eckenweber F., Zatcepin A. (2021). Microglial activation states drive glucose uptake and FDG-PET alterations in neurodegenerative diseases. Sci. Transl. Med..

[77] Jack C.R., Bennett D.A., Blennow K., Carrillo M.C., Dunn B., Haeberlein S.B., Holtzman D.M., Jagust W., Jessen F., Karlawish J. (2018). NIA-AA Research Framework: Toward a biological definition of Alzheimer’s disease. Alzheimers Dement..

[78] Mazziotta J.C., Toga A.W., Evans A., Fox P., Lancaster J. (1995). A probabilistic atlas of the human brain: Theory and rationale for its development. The International Consortium for Brain Mapping (ICBM). Neuroimage.

[79] Mazziotta J., Toga A., Evans A., Fox P., Lancaster J., Zilles K., Woods R., Paus T., Simpson G., Pike B. (2001). A four-dimensional probabilistic atlas of the human brain. J. Am. Med. Inform. Assoc..

[80] Bennett C.M., Wolford G.L., Miller M.B. (2009). The principled control of false positives in neuroimaging. Soc. Cogn. Affect. Neurosci..

[81] Liguori C., Chiaravalloti A., Sancesario G., Stefani A., Sancesario G.M., Mercuri N.B., Schillaci O., Pierantozzi M. (2016). Cerebrospinal fluid lactate levels and brain [18F]FDG PET hypometabolism within the default mode network in Alzheimer’s disease. Eur. J. Nucl. Med. Mol. Imaging.

